# Notch3 promotes 3T3‐L1 pre‐adipocytes differentiation by up‐regulating the expression of LARS to activate the mTOR pathway

**DOI:** 10.1111/jcmm.14849

**Published:** 2019-11-21

**Authors:** Yuxian Guo, Junyu Tan, Wei Xiong, Shuzhao Chen, Liping Fan, Yaochen Li

**Affiliations:** ^1^ The Central Laboratory of Shantou University Medical Cancer Hospital College Shantou China

**Keywords:** adipocytic differentiation, leucyl‐tRNA synthetase, mTOR pathway, Notch3, RNA‐seq, TMT proteomic analysis

## Abstract

Adipocytes constitute a major component of the tumour microenvironment. Numerous studies have shown that adipocytes promote aggressiveness and invasion by stimulating cancer cells proliferation and modulating their metabolism. Herein, we reported that Notch3 promotes mouse 3T3‐L1 pre‐adipocytes differentiation by performing the integrative transcriptome and TMT‐based proteomic analyses. The results revealed that aminoacyl‐tRNA_biosynthesis pathway was significantly influenced with Nocth3 change during 3T3‐L1 pre‐adipocytes differentiation, and the expression of LARS in this pathway was positively correlated with Notch3. Published studies have shown that LARS is a sensor of leucine that regulates the mTOR pathway activity, and the latter involves in adipogenesis. We therefore supposed that Notch3 might promote 3T3‐L1 pre‐adipocytes differentiation by up‐regulating LARS expression and activating mTOR pathway. CHIP and luciferase activity assay uncovered that Notch3 could transcriptionally regulate the expression of LARS gene. Oil Red staining identified a positive correlation between Notch3 expression and adipocytic differentiation. The activation of mTOR pathway caused by Notch3 overexpression could be attenuated by knocking down LARS expression. Altogether, our study revealed that Notch3 promotes adipocytic differentiation of 3T3‐L1 pre‐adipocytes cells by up‐regulating LARS expression and activating the mTOR pathway, which might be an emerging target for obesity treatment.

## 1 INTRODUCTION

Originally, adipose tissue was a relatively neglected component and just was regarded as an insulating and mechanically supportive tissue of energy storage and mobilization to meet some of the increased energy demand for peripheral organs.^1^, ^2^ With the deepen understanding, traditional views on adipose tissue have changed. Presently, adipose tissue is characterized to be as metabolically active organ, which can produce lipids or secrete adiponectin,^3^ leptin,^4^, ^5^ cytokines or adipokines.^6^

More recently, people have realized that working as a functional paracrine and endocrine tissue, adipose tissue plays multiple physiological roles in normal whole‐body metabolic homoeostasis,^7^ immune regulation^8^, ^9^, ^10^ and vascular function regulation,^11^ etc. In addition, the abundant evidence shows that enlarged adipose tissue, obesity, is closely associated with many different diseases, from gallstones, type 2 diabetes, hypertension and cardiovascular disease, even to cancer, which poses a great challenge to human health.^12^, ^13^ Accompanying obesity, the crosstalk between adipose and cancer‐prone cells may occur via obesity‐associated hormones, cytokines and other mediators and finally results in increased cancer risk and/or progression,^14^ which has started to gather more and more attention.^15^, ^16^

For example, Wolin et al reported that 20% of cancer patients was attributable to excess body weight.^17^ This is especially true in breast carcinomas because a mass of fatty tissue surrounds or extends throughout the breast.^2^ Thereby, it is very necessary to clarify which factors affect adipocyte differentiation and function.

Notch signalling that arises from the binding of the Notch receptor (Notch 1–Notch 4 in mammals) to its ligands (Delta‐like 1, Delta‐like 3, Delta‐like 4, Jagged 1 and Jagged 2)^18^ acts as a molecular gate to involve in a variety of cell‐fate choices by regulating the self‐renewal of stem cells and differentiation of progenitor cells. Upon ligand binding, Notch receptors are activated by serial cleavage events involving members of the ADAM protease family, as well as an intramembranous cleavage regulated by gamma secretase (presenilin). This intramembranous cleavage is followed by translocation of the intracellular domain on Notch to the nucleus, where it acts on downstream targets.^19^

Notch3 is important for the normal function and survival of vascular smooth muscle cells. The vertebrate Notch3 gene mutations have been shown to be involved in CADASIL.^20^, ^21^ Several researches have pointed out that Notch family members seem to have contradiction roles in adipocytes differentiation.^22^, ^23^ Herein, we provide solid evidence that Notch3 promotes adipocytic differentiation of 3T3‐L1 pre‐adipocytes by up‐regulating the expression of leucyl‐tRNA synthetase (LARS), which in turn activates the mTOR pathway.

## 2 MATERIAL AND METHODS

### 2.1 Cell culture and in vitro differentiation

Murine 3T3‐L1 cells were purchased from ATCC cultivated with DMEM (Life Technologies) containing 10% FBS at 37°C and 5% CO_2_. 3T3‐L1 cells differentiated into adipocytes as previously described, differentiation was induced by treating two days post‐confluent cells with DMEM containing 10% FBS and MDI mixture (1.0 μmol/L DEX, 0.5 mmol/L IBMX and 4 μg/mL insulin). On day three switched the media to DMEM with 10% FBS and 1 μg/mL Insulin. The medium was replaced every 2 days. Subsequent experiments were carried out in fully differentiated cells on day 8.

### 2.2 Gene knockdown and overexpression

We use siRNA to knockdown Notch3/LARS, in order to avoid off‐target effects we select no less than 2 siRNAs for each gene. We transfected the cells with an overexpressed plasmid in presence of liposome transfection reagent, and then screened the cells with puromycin to obtain the Notch3 stable‐overexpressing cells.

### 2.3 Oil Red staining

Differentiated 3T3‐L1cells were fixed with 10% formaldehyde at 4°C for 1 hour. After washing with 60% isopropanol, the fixed cells were stained with 0.4% Oil Red O in 3:2 (v/v) isopropanol/H_2_O for 30 minutes at room temperature and then rinsed three times with water. Lipid accumulation was observed under inverted light ZEISS microscope. The Oil Red O dye was eluted by 100% isopropanol, and the absorbance at 510 nm was detected with an ELISA reader.

### 2.4 Transcriptome profiling by RNA‐Seq

Total RNA of 3T3‐L1 cells with or without Notch3 knockdown was isolated using the TRIzol reagent (Invitrogen), according to the manufacturer's instructions. Equal amounts of total RNA from 3 negative control and 4 Notch3 knockdown samples were provided. Transcriptome sequence (RNA‐Seq) was performed by HuaDa Gene Company.

### 2.5 Tandem mass tagging

Cells of each group were harvested using ice‐cold PBS and collected in 1.5 mL Eppendorf tubes. The cells were homogenized in SDS protein lysate buffer by stirring for 30 minutes after sonication using centrifugation to remove cell debris and then cleaning up of SDC. Add n × 100 μL 2% TFA to the pellet to extract co‐precipitated peptides repeat twice. Merge all the supernatants, centrifuge at top speed for 20 minutes, transfer supernatant to a new tube. Use 30 kD ultra‐filter tube to filter out macro‐molecular. Peptide desalting for base‐RP fractionation: E Add 500 μL CAN to equilibrate C_18_ column then wash out ACN with 500 μL 0.1% FA 2 times, discard the washout. Load peptide solution to C_18_ column, let the solution slowly flow through the column. Peptides were fractionated to 120 fractions with high pH RPRP‐HPLC, and then combined to 8 fractions. LC‐MS/MS analysis was performed by a Q‐Exactive mass spectrometer (Thermo) equipped with a Nanospray Flex source (Thermo).

### 2.6 Validation of transcriptome analyses using quantitative PCR

Total RNA (1 μg) was reverse‐transcribed using PrimeScript™ RT reagent Kit with gDNA Eraser (Takara Biomedical Technology) Quantitative PCR was then used to confirm the significantly altered genes revealed in the RNA‐Seq analyses. The qRT‐PCR primers to determine the target gene expression levels were shown in Table S1. Each quantitative real‐time PCR was carried out in triplicate with SYBR Green Real‐time PCR Master Mix (Roche). The expression levels were normalized to the 18S in each sample.

### 2.7 Gene set enrichment analysis (GSEA)

Gene set enrichment analysis was performed as described by Lin et al.[24] A list of genes enriched in this study was given in Table S2.

### 2.8 Protein extraction and Western blotting

To detect protein levels, 3T3‐L1 cells were obtained with ice‐cold RIPA Lysis buffer (Beyotime). Protein samples were quantified using a bicinchoninic acid kit (Beyotime). Western blot was performed by using an appropriated dilution of the primary and the secondary antibodies in Table S3. Detection of GAPDH with a specific antibody (ZSGB‐BIO) was used as a protein loading control. Finally, PVDF membranes were scanned using the Quantity One Imaging system (Bio‐rad). The relative expression of target protein was normalized to that of GAPDH. Every experiment was repeated three times. All values are presented as mean ± SEM.

### 2.9 Chromatin immunoprecipitation (ChIP)

Chromatin immunoprecipitation was performed as previously described, with minor modifications.[25] To determine whether the Notch3 regulates the expression of LARS, cross‐linked chromatin was sheared by sonication using a Biosafer250‐88 Ultrasonic homogenizer (Biosafer) four times for 4 seconds and an interval of 9 seconds with a microtip in a 1.5‐mL tube. The supernatant from the irrelevant antibody served as a positive control (‘input’, 1% of the ChIP material). Chromatin was immunoprecipitated with Notch3 antibody. Immunoglobulin G (IgG) was used as a negative control. Protein A/G agarose beads were added to antibody/chromatin complexes and incubated overnight at 4°C. The protein A/G agarose‐antibody/chromatin complex was resuspended in wash buffer and centrifuged to collect the protein/DNA complex. Protein/DNA crosslinks were reversed to obtain free DNA. Purified, immunoprecipitated DNA was analysed by semi‐quantitative PCR. ChIP primer sequences are provided in Table S1.

### 2.10 Statistical analysis

Statistical analysis was performed using SPSS 18.0 software. Results were presented as mean ± SEM. Statistically significant differences were calculated using a Student's *t* test. A *P*‐value of .05 was considered significant.

## 3 RESULTS

### 3.1 Establishments of the stable 3T3‐L1 pre‐adipocytes cell line overexpression Notch3 and transient 3T3‐L pre‐adipocytes knocking down Notch3

**Figure 1:**
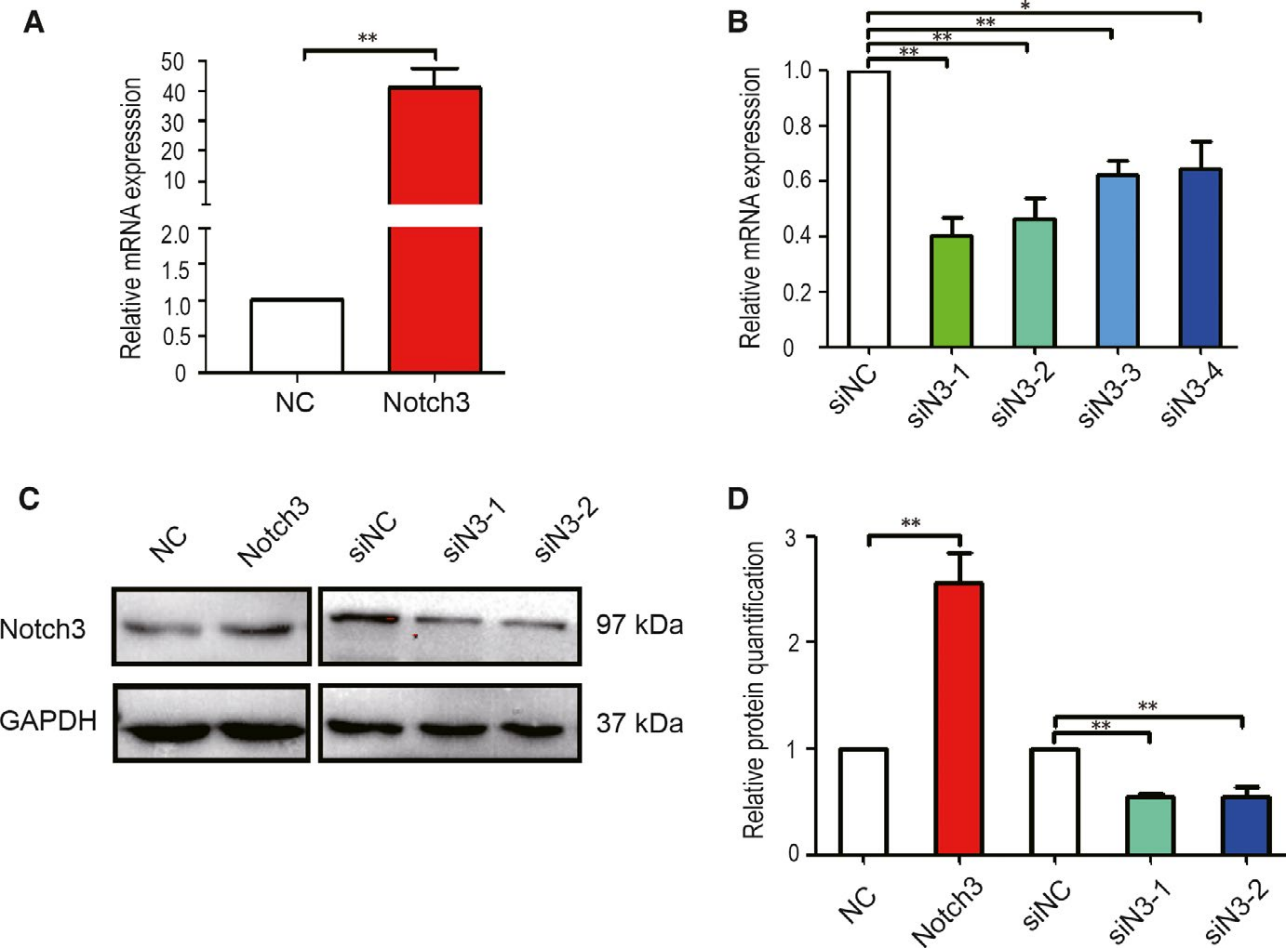
Validation of Notch3 overexpression and Notch3 knockdown at mRNA and protein levels. A, Notch3 gene expression in RNA level after transfected with Notch3 overexpression plasmid. B, Notch3 gene expression in RNA level after transfected with small interfering RNA. C, Notch3 gene expression in protein level. D, Quantification of Notch3 gene expression in protein level. The experiment was repeated more than three times data are mean ± standard error of the mean (SEM) of three independent experiments*P < .05, **P < .01 indicates the mean value is significantly different from that of the control

To elucidate the effect of Notch3 on adipocytic differentiation, the stable 3T3‐L1 pre‐adipocytes cell line overexpression Notch3 was established by infecting the cells with Notch3 overexpression lentivirus. The total RNA and the lysate were isolated and used to verify the overexpression efficiency. The results showed that the expression level of Notch3 was sharply up‐regulated in 3T3‐L1/Notch3 cells, which was about 40 times higher than that in the control group (Figure 1A). The up‐regulated Notch3 expression was also supported by Western blot assay (Figure 1C). As shown in Figure 1D, quantitative analysis of Western blot results showed that the expression of Notch3 protein in 3T3‐L1/Notch3 cells was about 2.5 times higher than that in control cells. By contrast, we used RNA interference (RNAi) technique to investigate the effect of knocking down the expression of the Notch3 gene in 3T3‐L1 on the adipocytic differentiation. The scrambled sequence was used as negative control. The knocking down efficiency was compared by qRT‐PCR and Western blot. Compared with siNC group, RT‐PCR result uncovered that after transfection of these interfering sequences, the Notch3 mRNA expression in all four group was significantly inhibited, but the knocking down efficiencies in siN3‐1 and siN3‐2 groups were better (Figure 1B). Likewise, Western blot assay showed similar results as real‐time RT‐PCR (Figure 1C,D).

### 3.2 RNA‐seq analysis and qRT‐PCR verification

**Figure 2:**
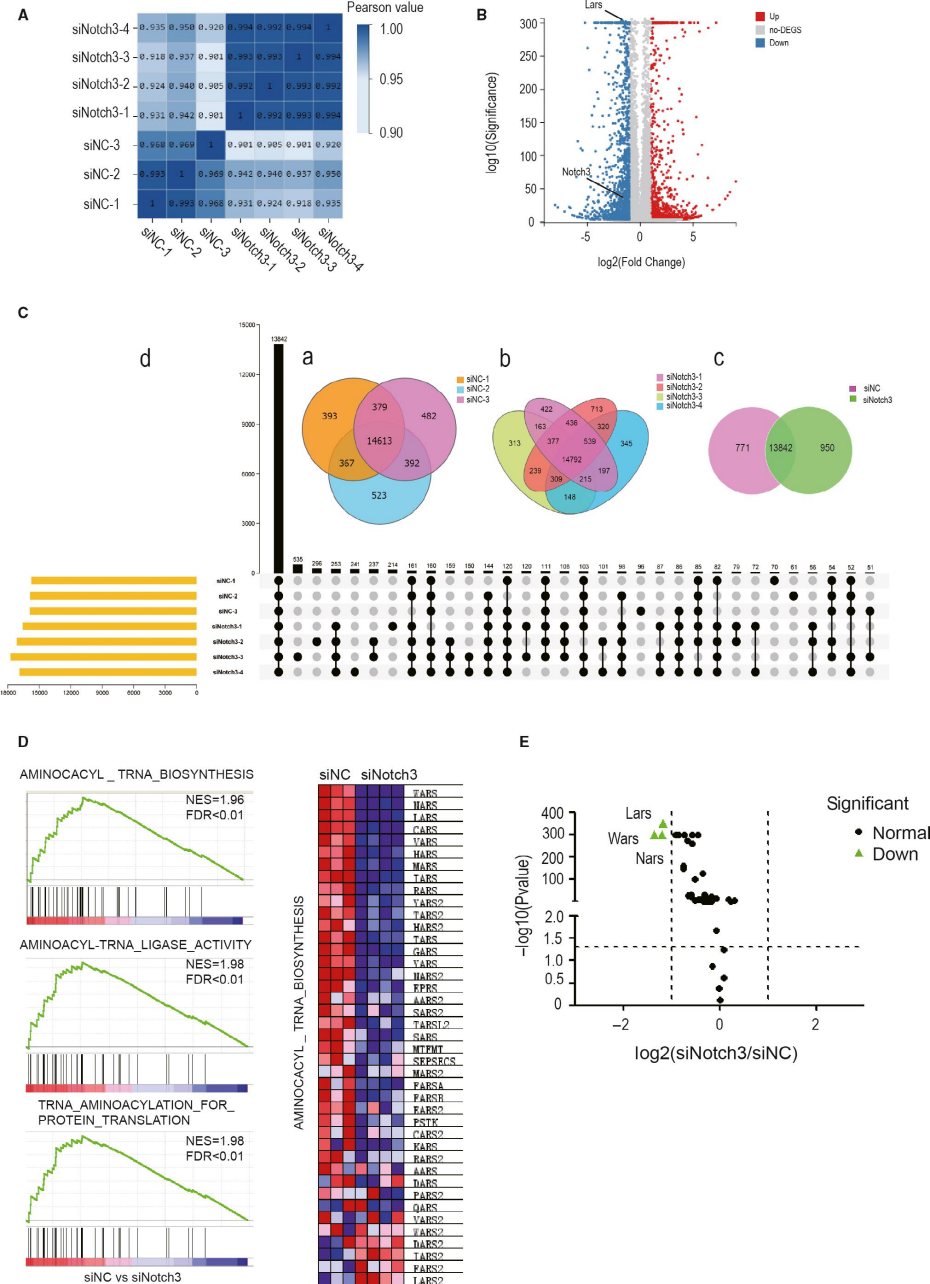
RNA‐seq data for control and Notch3 knockdown samples. A, The Pearson correlation coefficients of all gene expression between each two samples the higher the correlation coefficient was, the more similar the gene expression level was. B, The volcano plot shows the up‐ (red) or down‐regulated (blue) genes between Notch3 knockdown and the control groups. C, Venn diagram and UpSet plot showing the overlap of control samples and Notch3 knockdown samples. D, Gene set enrichment analysis（GSEA）of 3T3‐L1 pre‐adipocytes after Notch3 interference. The GSEA results showing that the LARS relative gene sets ‘AMINOACYL‐TRNA BIOSYNTHESIS’, ‘AMINOACYL‐TRNA_LIGASE_ACTIVITY’ and ‘TRNA_AMINOACYLATION_FOR PROTEIN_TRANSLATION’ were enriched in Notch3 knockdown condition. Heat map of core enrichment genes in the gene set AMINOACYL‐TRNA BIOSYNTHESIS. The GSEA software was used to calculate the enrichment levels. E, Volcano map of core enrichment genes in the gene set AMINOACYL‐TRNA BIOSYNTHESIS

To ascertain the molecular mechanism the influence of Notch3 on adipocytic differentiation, RNA‐seq analysis was performed. The sequencing data from three 3T3‐L1/siN3 as well as four 3T3‐L1/siNC total RNA samples were used to analyse the technical reproducibility of RNA‐seq. We calculated the pairwise Pearson correlation coefficients of FPKM values between samples, and the heat map was produced. The Pearson correlation coefficients were shown in the Figure 2A and Table S4. The results showed these samples were highly correlated and did not have larger difference in the same group, and experimental samples were separated from control samples.

As shown in the Venn diagram (Figure 2C), 15 752, 15 866 and 15 895 genes were detected in siNC‐1, siNC‐2 and siNC‐3, respectively (Figure 2C‐a), while 16 556, 17 141, 17 725 and 16 865 genes were detected in siN3‐1, siN3‐2, siN3‐3 and siN3‐4, respectively (Figure 2C‐b). Of these detected gens, 14 613 genes were co‐expressed in three control samples, and 14 792 genes were co‐expressed in four Notch3 knocking down samples. A total of 13 842 co‐expressed genes were found between the control and experimental groups (Figure 2C‐c and d).

Comparison of 13 842 co‐expressed genes revealed 899 genes up‐regulated by twofold or more and 1541 genes down‐regulated by 0.5‐fold or more (*P* ≤ .001). These differentially expressed genes were shown in the volcano plot, in which the red colours represented the up‐regulated genes, and the blue colours exhibited the down‐regulated genes (Figure 2B).

Gene set enrichment analysis (GSEA) was performed by using the computational method (http://software.broadinstitute.org/gsea/index.jsp). Based on the criterion, *P*‐value < .001 and FDR < 0.1, 598 signalling pathway was screened out. The top enriched pathways were aminoacyl‐tRNA biosynthesis signalling pathway (NES = 1.96, *P*‐value = 0, FDR = 0.001478998), aminoacyl‐tRNA ligase activity, tRNA aminoacylation for protein translation and so on. Especially, we paid attention to the AMINOACYL‐TRNA_BIOSYNTHESIS pathway. A number of 41 genes were enriched in this pathway (Figure 2D). The heat map and the volcano plot showed the expression level of every gene enriched in this pathway (Figure 2D). To sum up, Notch3 knockdown may affect the differentiation of 3T3‐L pre‐adipocytes via aminoacyl‐tRNA biosynthesis pathway.

### 3.3 Proteomic analysis and Western blot verification

**Figure 3:**
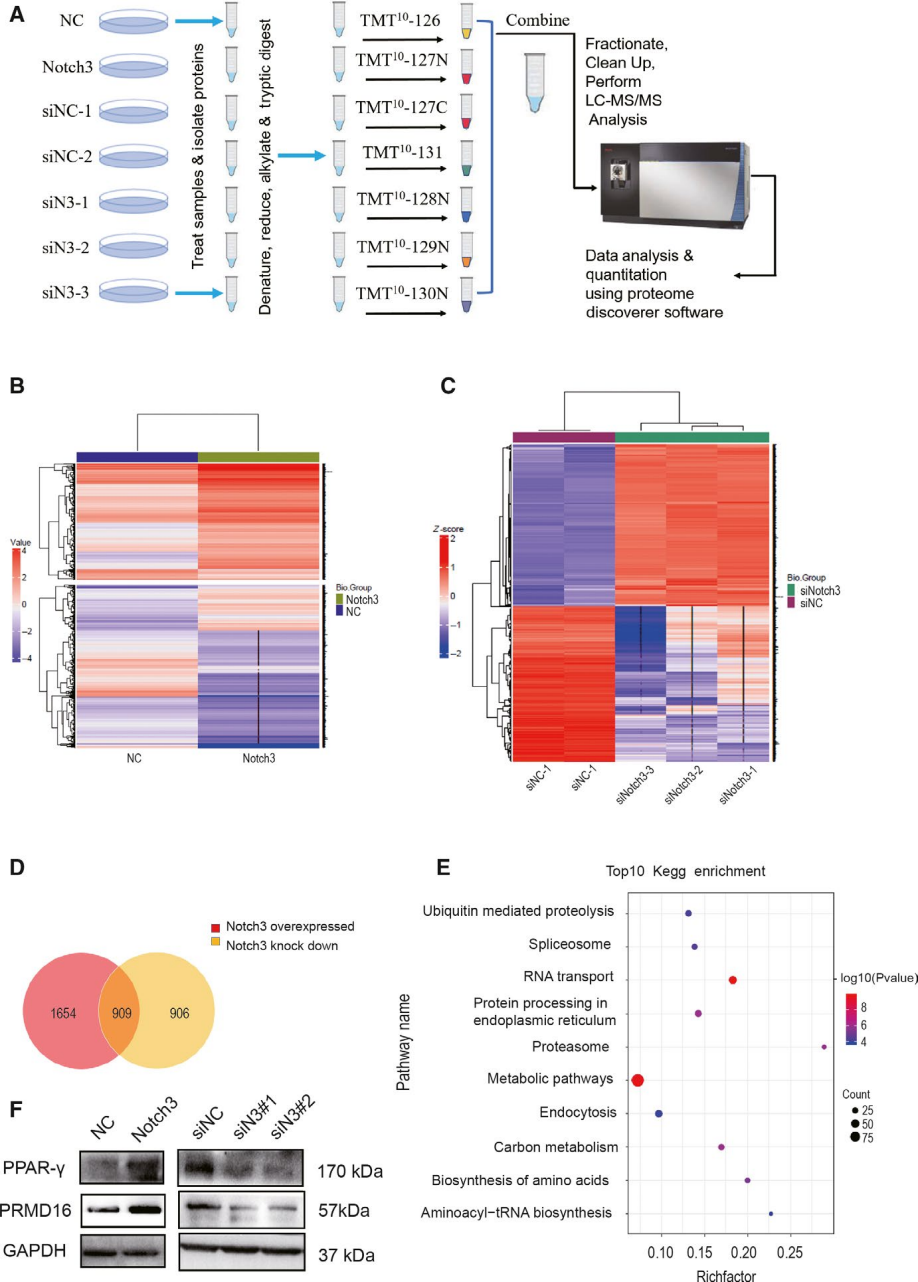
Proteins TMT quantification of 3T3‐L1cells with different treat. A, TMT experimental flow chart and samples are labelled with the TMT Reagents and then mixed before sample fractionation and clean up. Peptide fractions are analysed by high‐resolution Orbitrap LC‐MS/MS before data analysis to identify peptides and quantify reporter ions relative abundance. (B, C) Clustering heat map of all significant proteins. Use Pearson's distance if there are 3 or more samples or Euclidean's distance if not. Miss values are indicated with ‘−’. The colour scale bar locates in the left, and red and blue indicate increased and decreased levels of the identified proteins, respectively. D, Venn diagram showing the overlap of Notch3 overexpressed and Notch3 knockdown samples. E, KEGG enrichment analysis bubble diagram showed top10 pathways in a total number of 40 enriched pathways according to FDR < 0.05. (F) Western blot results of PPARγ and PRMD16 were used to verify the dependability of TMT quantitative proteomics analysis

To confirm the molecular mechanism the influence of Notch3 on adipocytic differentiation, we performed the deeper investigations in total 3T3‐L1/siN3 and 3T3‐L1/siNC lysates as well as 3T3‐L1/Notch3 versus empty vector control 3T3‐L1/NC lysates using a multi‐plex tandem mass tagging (TMT) isobaric labelling quantitative proteomics comparisons. TMT increases sample throughput, enables relative quantitation of up to 10 different samples and increases multi‐plex capability results in fewer missing quantitative values. TMT‐10plex can concurrent MS analysis of our 7 samples. Procedure summary for MS experiments with 10‐plex TMT Reagents are shown in Figure 3A. Lysates were combined and purified for QE Orbitrap liquid chromatography‐tandem mass spectrometry (LC‐MS/MS) analysis. The results were then searched against a Mus musculus protein database for identification. After hierarchical clustering of expressional values of differentially proteins, Notch3 overexpression and siN3 samples were completely separated from the 3T3‐L1/NC and siNC samples, respectively, indicating that the expression pattern screening the differentially proteins were significantly characteristic (Figure 3B,C).

The Western blot analysis served to validate the results by TMT quantitative proteomics method. The results exhibited that the expression of Notch3, PPARγ and PRMD16 in the Notch3 overexpression group was significantly higher than that in the control group, whereas the expression of Notch3 in the Notch3 knockdown group was significantly lower than that in the control group (Figure 3F). In summary, these data emphasize that the results of Western blot for these proteins are also consistent with the proteomic data. Venn diagram showing that 2563 differential proteins were detected in 3T3‐L1/N3 overexpression *versus* 3T3‐L1/NC, and differential 1815 proteins were detected in 3T3‐L1/siN3 versus 3T3‐L1/siNC group according to the following criteria: the fold change was more than twofold down (or up). In total, 909 differential proteins (with a minimum of 2 unique peptides identified per protein) were found along with overexpressing or knocking down Notch3. Of the 909 differential proteins, 718 differential proteins were relative to Notch3 very well, their expression varied with Notch3 overexpression or knockdown, but other 191 differential proteins did not (Figure 3D). Next, the 718 differential proteins were the focus in this study.

KEGG enrichment analysis was carried out. The bubble diagram showed top10 pathways in a total number of 40 enriched pathways according to FDR < 0.05 (Figure 3E), they were ubiquitin‐mediated proteolysis, spliceosome, RNA transport, protein processing in endoplasmic reticulum, proteasome, metabolic pathway, endocytosis, carbon metabolism, biosynthesis of amino acid and aminoacyl‐tRNA biosynthesis. Integrative analysis of transcriptome and proteome revealed that aminoacyl‐tRNA biosynthesis pathway was enriched either at transcription level or at protein level accompanied by Notch3 overexpression or knockdown. Altogether, these data provide the new insights that aminoacyl‐tRNA biosynthesis may be the important pathway for Notch3 to regulate adipogenesis of 3T3‐L1 cells.

### 3.4 There is a positive correlation between Notch3 expression and adipocytic differentiation

**Figure 4:**
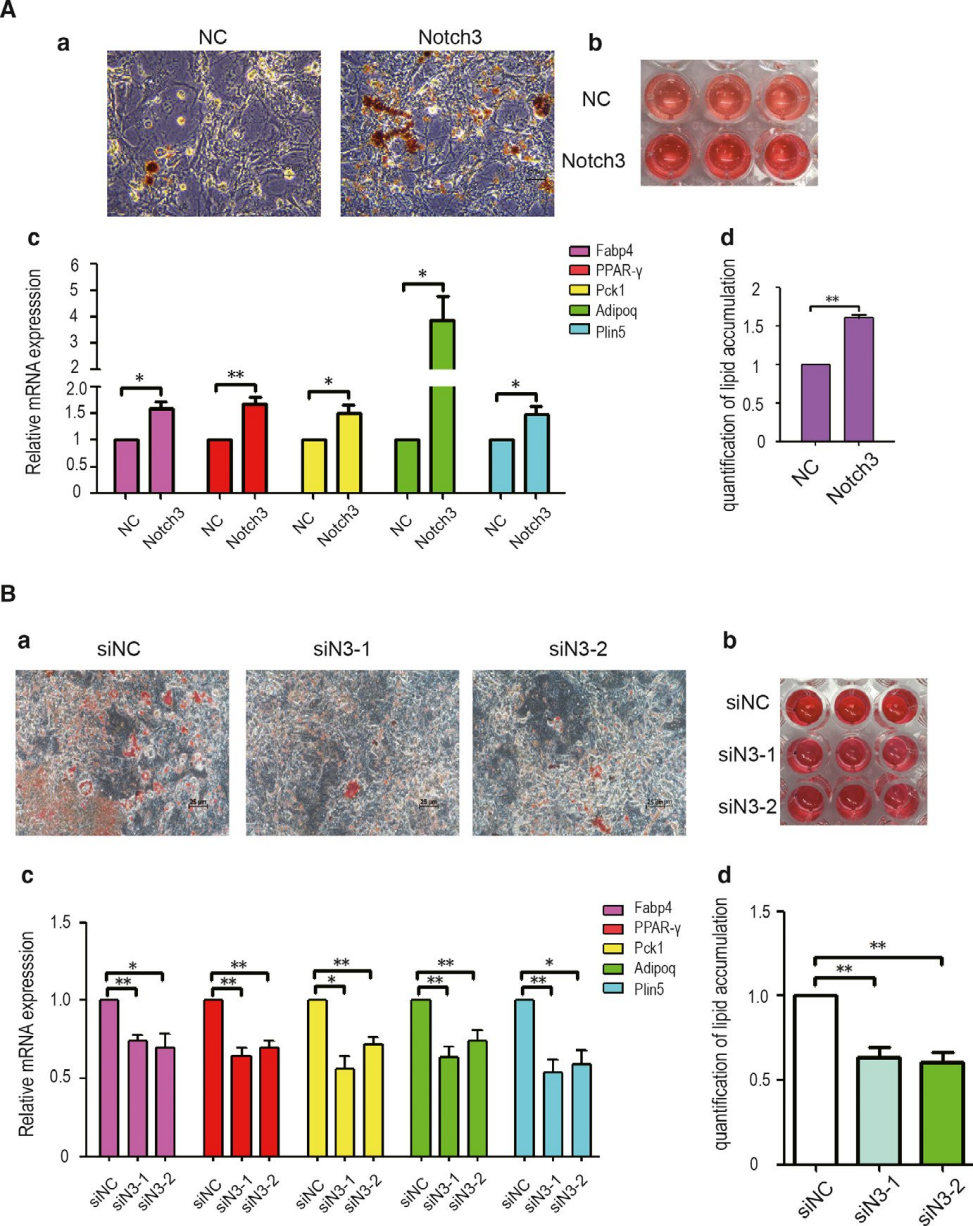
Notch3 acts as a positive regulator of adipocyte differentiation in 3T3‐L1 cells. (A‐a) Graphs show bright‐field microscopy of differentiated 3T3‐L1 adipocytes stained by Oil Red O for control and Notch3 overexpression cells. Scale bar indicates 25 μm. (A‐b) The Oil Red O dye was eluted by using 100% isopropanol. (A‐c) RT‐qPCR assay of the expression of lipid metabolism‐related genes Fabp4, PPARγ, Pck1, Adipoq and Plin5. (A‐d) the absorbance at 510 nm was determined using an ELISA reader. B, Graphs for negative control and Notch3 knockdown group. Data are means ± standard error of the mean (SEM) of three independent experiments *P < .05, **P < .01 indicates the mean value is significantly different from that of the control

To further determine whether Notch3 affects adipocyte differentiation in 3T3‐L1 pre‐adipocytes cells, the 3T3‐L1/N3 and 3T3‐L1/siN3 cells were induced by induction medium (0.5 mmol/L IBMX, 1.0 μmol/L DEX and 4 μg/mL insulin) after 2‐day post‐confluent. Cell RNA and protein were determined on day 3, and Oil Red O staining was performed on day 8. In addition, Oil red staining was extracted with 100% isopropanol and observed in 96 well plates. As presented in Figure 4A‐a, Notch3 overexpression induced differentiation, as detected using an Oil Red O assay, and the number of lipid droplets increased markedly in the Notch3 overexpression group compared with the control group (magnification 200×). The colour of Notch3 overexpression group was darker than that of control group (Figure 4A‐b). The absorbance at 510 nm of Notch3 overexpression group was higher than that in control group (Figure 4A‐d). RT‐qPCR assay also indicated that the expression of lipid metabolism‐related genes, such as Fabp4, PPARγ, Pck1, Adipoq and perilipin 5 (Plin5), was increased significantly in 3T3‐L1/N3 cells compared with the control group (Figure 4A‐c). By contrast, the number of lipid droplets decreased markedly in 3T3‐L1/siN3 cells when compared with 3T3‐L1/siNC cells (magnification 200×) (Figure 4B‐a). The colour of Notch3 knocking down group was lighter than that of control group (Figure 4B‐b). Likewise, the absorbance at 510 nm of Notch3 knocking down group was lower than that of control group (Figure 4B‐d). RT‐qPCR assay also indicated that the expression of Fabp4, PPARγ, Pck1, Adipoq and perilipin 5 (Plin5) was significantly decreased in 3T3‐L1/siN3 cells compared with the control group (Figure 4B‐c). To further verify the effect of Notch3 on the adipocytic differentiation, the rescue experiments were carried out. LARS gene was knocked down with siRNA in Notch3‐overexpressing 3T3‐L1 cells. The results from oil Red O staining showed that the number of lipid droplets decreased markedly in the LARS knockdown groups compared with the control group (magnification 200×) (Figure S1A). The colour of the LARS knockdown groups was lighter than that of control group (Figure S1B). The absorbance at 510 nm of LARS knockdown groups was lower than that in control group (Figure S1C). RT‐qPCR assay also indicated that the expression of lipid metabolism‐related genes, such as Fabp4, PPARγ, Adipoq and Plin5, was decreased significantly in siLARS groups compared with the control group (Figure S1D). Altogether, Notch3 promotes adipocytic differentiation of mouse 3T3‐L1 cells.

### 3.5 Notch3 promotes adipocytic differentiation of 3T3‐L1 cells by up‐regulating the expression of LARS and activating the mTOR pathway

**Figure 5:**
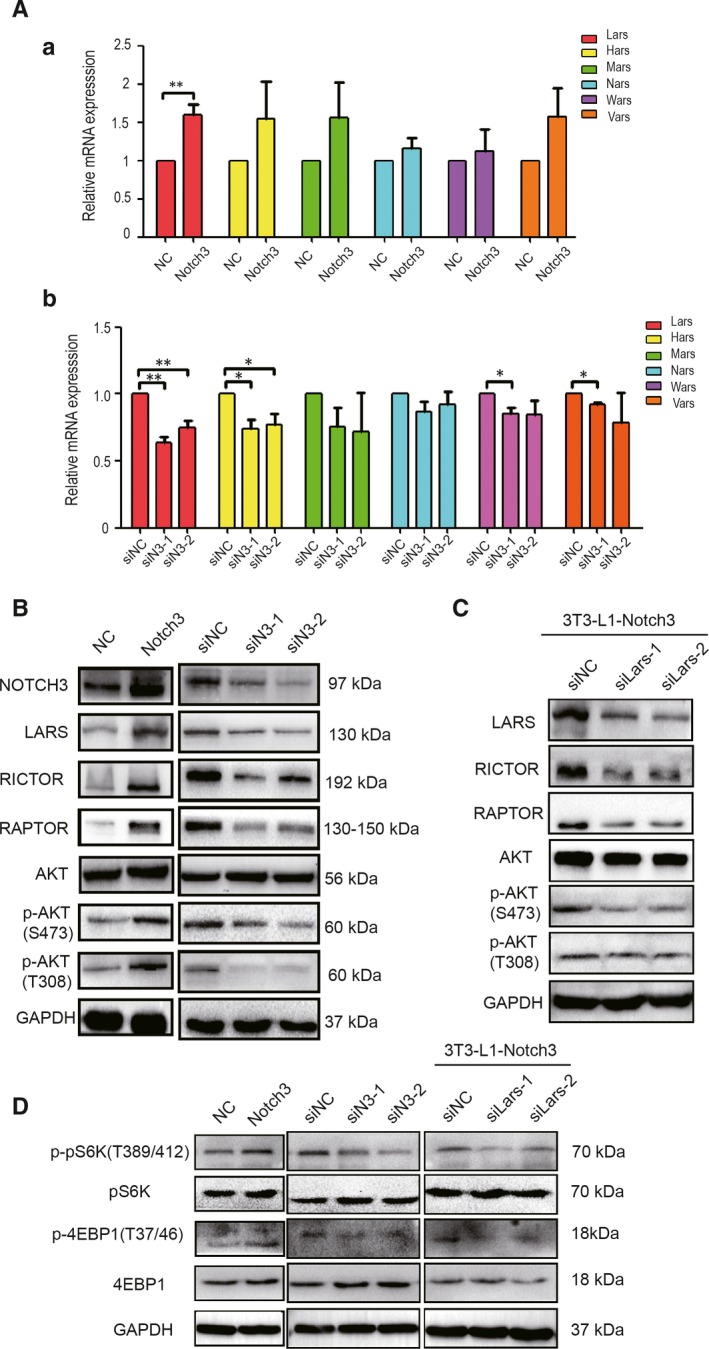
Detection of mTOR pathway‐related genes after Notch3 gene expression level change. A, RT‐PCR detect AMINOACYL‐TRNA BIOSYNTHESIS pathway members expression in RNA level after Notch3 gene expression change. B, Western blot was analysed for LARS, Rictor, Raptor, AKT, p‐AKT(S473) and p‐AKT(T308) protein expression LARS (C) The rescue experiment showed that the expression of the members in mTOR signalling pathway were decreased after interfering LARS expression in Notch3 stable overexpression 3T3‐L1 cells. D, The downstream of mTOR signalling pathway pS6K, 4EBP1 and their phosphorylation forms showed that the mTOR signalling pathway was affected by Notch3 and LARS. Data are mean ± standard error of the mean (SEM) of three independent experiments *P < .05, **P < .01 indicates the mean value is significantly different from that of the control

To ascertain which genes in the aminoacyl‐tRNA biosynthesis pathway played key role in differentiation of 3T3‐L1 pre‐adipocytes, the expression levels of LARS, HARS, MARS, NARS, WARS and VARS were detected by real‐time PCR. The results showed that only the expression of LARS varied very well along with Notch3 increase or decrease, suggesting that LARS mediates the effects of Notch3 (Figure 5A‐a and b). These results were also supported by Western blot (Figure 5B). To ascertain the effects of Notch3 and LARS on adipocyte differentiation, we detected the expression levels of Notch3 and Lars mRNA at day 0, 2, 4, 6 and 8 during adipocyte differentiation. The results showed that Notch3 expression increased at day 2 followed by a trend of continuous increase at day 4 and day 6, then fell back to the original level at day 8 (blue colour) when compared with the beginning of the differentiation (day 0). Similarly, LARS showed an increased trend at day 2 and day 4 and increased significantly at day 6, then decreased at day 8 accompanied with the increase or decrease of Notch3 expression (red colour). Of note, the expression of LARS at day 8 was still significantly higher than that at day 0 (Figure S2). The published studies reported that LARS plays a key role in regulating mTOR pathway, and furthermore, mTOR pathway may play a key role in adipogenesis, we therefore inferred that Notch3 might affect the mTOR pathway via regulating LARS expression.

Moreover, the expression levels of some proteins in mTOR pathway were detected after Notch3 overexpression or knockdown. The Western blot results indicated that the protein expression levels of Rictor and Raptor increased or decreased after Notch3 overexpression or knockdown, respectively. In addition, two phosphorylation forms of AKT, p‐Akt (T308) and p‐Akt (S473), are active forms, whose expression increased or decreased along with Notch3 overexpression or knockdown, respectively, while no significant change was observed in total AKT expression level (Figure 5B). In order to better obtain the evidence that Notch3 affected adipocytic differentiation via LARS mediating mTOR pathway. The downstream molecules of mTOR pathway, pS6K and 4EBP1, were tested. There were no significant changes in total pS6K and 4EBP1 expression level; however, their phosphorylation form changed significantly with Notch3 overexpression or knockdown. The results from rescue experiments also showed that the expression of the members in mTOR pathway was also decreased after interfering the expression of LARS gene in stably Notch3‐overexpressing 3T3‐L1 cells (Figure 5C). Collectively, the data reflect that Notch3 can regulate the activity of mTOR pathway via LARS.

### 3.6 Notch3 transcriptionally regulate LARS expression

**Figure 6:**
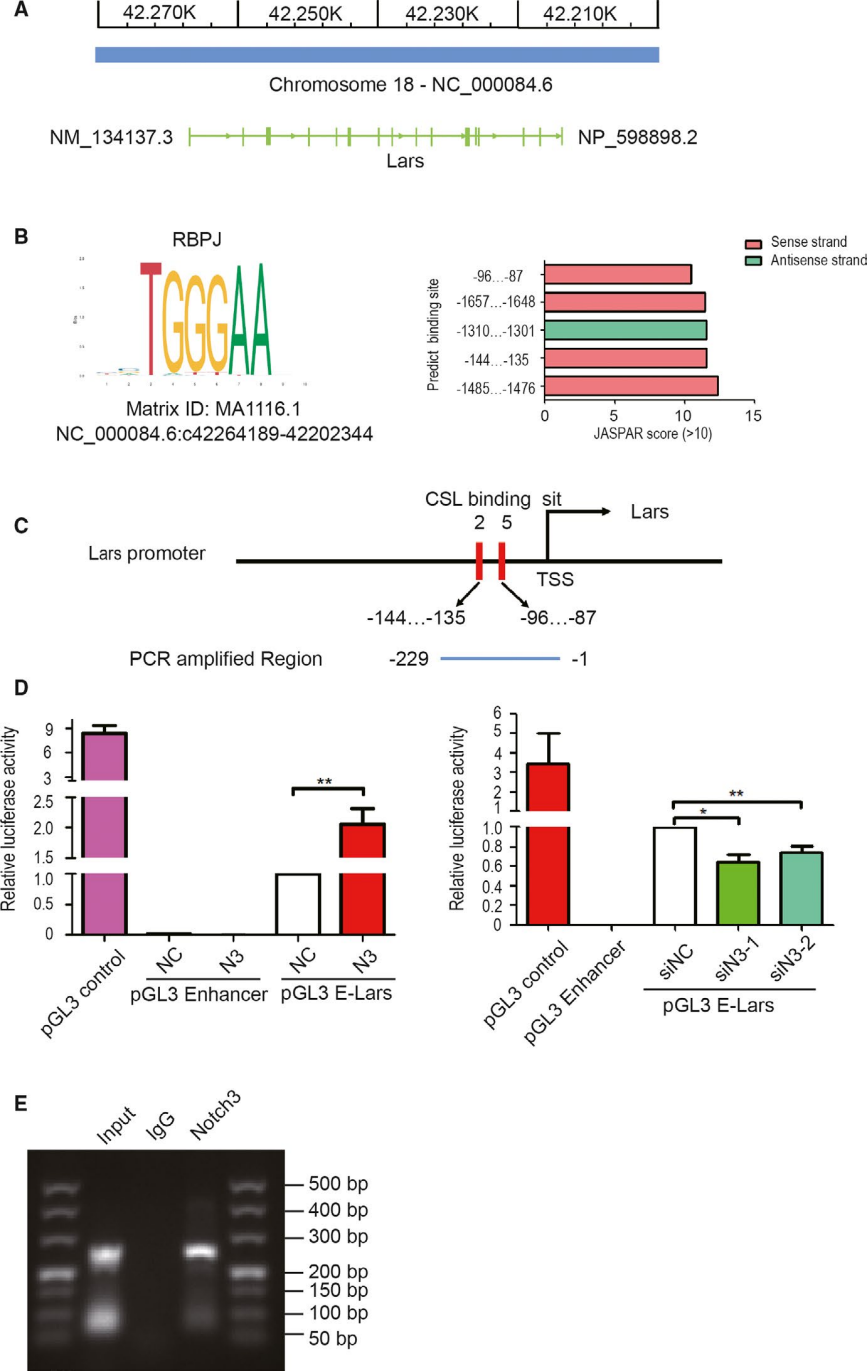
Notch3 transcriptionally regulates the expression of LARS in 3T3‐L1 cells. A, The LARS gene information on the chromosome 18. B, Top five of 18 predicted notch3 CSL binding site in LARS gene promoter and the core sequence of RBPJ. C, Designed primers to amplify a short 228 bp fragment containing two of the predicted binding sites to construct the reporter gene. D, Reporter gene assay illustrated that Notch3 can combined with the predicted LARS promoter region. E, ChIP assay confirmed Notch3 combination with the LARS's promoter region. Data are mean ± standard error of the mean (SEM) of three independent experiments* *P* < .05, ** *P* < .01 indicates the mean value is significantly different from that of the control

To ascertain the mechanism that Notch3 regulated LARS, we analysed the location LARS gene on the chromosome, as well as the sequence of promoter and first non‐encoding exon of LARS (−2000 ~ 105 bp) by Jaspar online tool (http://jaspar.genereg.net/). The result showed that the coding strand of LARS gene corresponds to the chromosome's reverse strand (Figure 6A). Jaspar analysis uncovered that five putative core sequence TGGGAA of RBP‐jk binding sites in promoter regions of LARS gene were observed, suggesting that transcription factor Notch3 might act as an upstream inducer of LARS, most likely in a direct manner. The locations on the strand and scores were shown in Figure 6B. We chose the region from −229 ~ −1 to study, in which two RBP‐jk binding sites with higher score are adjacent closely, −144 bp ~ −135 bp and −96 bp ~ −87 bp (Figure 6C).

We further constructed a luciferase reporter vector contained these two RBP‐jk binding site of LARS promoter. A dual‐luciferase reporter assay was carried out by transient co‐transfection with the plasmid‐containing LARS promoter, as well as a Renilla luciferase reporter vector with or without co‐expression of the N3ICD expression vector. LARS promoter activity increased 2.06‐fold in the 293T cells with Notch3 overexpression (Figure 6D left, *P* < .01). By contrast, the LARS promoter activities, respectively, showed decrease of 35% and 25% in siN3‐1 and siN3‐2 cells (Figure 6D right; *P* < .01 or .05). These data suggest that Notch3 activates LARS expression by binding to the RBP‐jk‐binding site of the LARS promoter. The conclusion was also supported by chromatin immunoprecipitation (ChIP) assay performed using an anti‐Notch3 antibody (Figure 6E). Obviously, binding to the region in the LARS promoter containing RBP‐jk binding motifs was clearly detected in Notch3 antibody pull‐down group when compared with the negative IgG control group. To sum up, Notch3 transcriptionally regulates LARS expression.

## 4 DISCUSSION

3T3‐L1 is a cell line derived from murine Swiss 3T3 cells that differentiate into an adipocyte‐like cell under appropriate conditions, which can be tracked by Oil Red O staining to monitor lipid accumulation. Thus, 3T3‐L1 differentiation is an economical and convenient way for us to generate adipocyte‐like cells and to study certain molecular mechanism regulating adipogenesis.

In this study, we investigated the effect of Notch3 on 3T3‐L1 pre‐adipocytes differentiation as well as the associated molecular mechanism. We found that Notch3 expression positive correlated with adipogenesis (fat formation). In detail, Notch 3 overexpression increased the adipogenesis, on the contrary, knocking down Notch3 decreased the adipogenesis in 3T3‐L1 adipocytes.

Then, what is the molecular mechanism? To settle this question, integrative RNA‐Seq and TMT‐based proteomic analysis were performed. The transcriptome, which is assessed by RNA‐seq, is widely considered to be the most mature omics technology with a long measure and the greatest depth of coverage.^26^, ^27^ This approach is also advantageous when the protein products of genes are either recalcitrant to proteomic analysis or of low abundance, which can result in their under‐representation in some conditions.^28^ The proteomic analysis is widely used in the analyses of differential expression of proteins in order to understand the changes of protein expression between experimental and control group. The proteins are the ultimate executors of cellular function. A fine change in proteins may affect the overall function of cells. The proteomic approach can precisely provide useful information at an index of levels.^29^, ^30^, ^31^ In the current study, the transcriptome analysis revealed that 13 842 co‐expressed genes were found between the control and experimental group. The proteomic analysis showed that 3034 proteins were detected when Notch3 overexpressed, and 2690 proteins were detected in N3 knockdown group. Unsatisfactorily, the correlation between the proteome and transcriptome was poor. These data suggest that transcriptome analysis was more comprehensive than the proteomic analysis. This finding may due to two reasons. The first is the different lifespans of mRNA compared with proteins or the transport of proteins and post‐transcriptional modification. Translation, the synthesis of proteins by ribosomes using an mRNA template, is a fundamental process in biology. The second probability is that adipocyte characterized by a lot of lipid accumulation, production of leptin as well as adipocyte marker expression, which may lead to the undetectable low abundance proteins. These differences may explain the discrepancies in the transcriptomic and proteomic data in our study. Collectively, RNA‐seq analysis is a particularly useful tool for exploring the adipogenesis, such as 3T3‐L1 pre‐adipocyte differentiation.

Integrative analyses revealed that aminoacyl‐tRNA_biosynthesis pathway was significantly affected by Nocth3 change during 3T3‐L1 pre‐adipocytes differentiation, and the expression of LARS in this pathway was positively correlated with Notch3. Aminoacylation of transfer RNAs is catalysed by an ancient group of 20 enzymes (one for each amino acid) known as aminoacyl‐tRNA synthetases (AARSs), establishing the rules of the genetic code and catalysing an early step in protein synthesis.^32^ Kim et al showed that leucyl‐tRNA synthetase (LRS) plays a critical role in amino acid‐induced mTORC1 activation by sensing intracellular leucine concentration and initiating molecular events leading to mTORC1 activation.^33^ In short, LARS is a sensor of leucine that regulates the mTOR pathway activity. Importantly, the Rictor‐mTOR complex has also been reported to regulate Akt activation directly and facilitated Thr308 phosphorylation by PDK1.^34^ Moreover, the ser/thr kinase Akt (or protein kinase B/PKB) plays an essential role in adipocyte differentiation. Constitutively active Akt can promote the differentiation of 3T3‐L1 cells into adipocytes.^35^ At least two downstream branches of Akt signalling have been implicated in the regulation of PPARγ expression and adipocyte differentiation. One is that Akt‐mediated inhibition of FOXO1 is one mechanism by which Akt induces PPARγ and subsequent adipocyte differentiation. Another major signalling branch downstream of Akt results in activation of the mammalian target of rapamycin (mTOR), a critical regulator of mRNA translation and cell growth.^36^ In our current study, we found the expression of LARS, active forms of Akt and its downstream molecules mTORC1, Rictor and Raptor, increased with Notch3 overexpression, but also down‐regulated accompanied by Notch3 knockdown. Obviously, Notch3 influenced adipocyte differentiation might be via LARS mediated mTORC1.

Then, we ask whether and how Notch3 regulated LARS expression. We analysed LARS gene and its promoter. Usually, a gene can live on a DNA strand in one of two orientations. The gene is said to have a coding strand (also known as its sense strand), and a template strand (also known as its antisense strand). For 50% of genes, its coding strand will correspond to the chromosome's forward strand, and for the other 50% it will correspond to the reverse strand. Very interesting, we found that LARS gene corresponds to the reverse strand. The real meaning that the coding strand of a gene corresponds to the chromosome's reverse strand is remain largely unknown, which is required further study. Next, we noticed that the promoter contained five classical RBP‐jk binding regions which can be recognized and regulated by the NOTCH family, indicating the possibility of the regulation of LARS by Notch3.

By manipulating Notch3 expression, we demonstrated that Notch3 could regulate the expression of LARS in both protein and mRNA levels in 3T3‐L1 cells. Moreover, we have proved that Notch3 could bind to the RBP‐jk region and promote the promoter activity by chromatin immunoprecipitation and Luciferase assays. All these data suggest that Notch3 could transcriptionally regulate LARS expression in 3T3‐L1 pre‐adipocyte, which reveals a new aspect of the leucyl‐tRNA synthetase regulation mechanism involving adipogenesis role of Notch3.

In summary, in this study, we found Notch3 transcriptionally regulates LARS expression, and the latter plays a critical role in amino acid‐induced mTORC1 activation to control adipogenesis. We have provided compelling genetic, proteome, cellular and molecular evidence to demonstrate that Notch signalling promotes adipogenesis. According to these facts, we can conclude that LARS is a pivotal intermediate molecule to mediate the activity of mTORC1 pathway and adipogenesis. Particularly, recent studies have also been shown the aminoacyl‐tRNA synthetases to be promising targets in the development of antimicrobial agents^37^ as well as in therapeutics against cancers and other diseases.^32^ Then, LARS might serve as a potential anti‐obesity target and that the nutritional signalling pathway may provide a valuable anti‐obesity strategy for further investigation.

## CONFLICT OF INTEREST

The authors declare no potential conflicts of interest.

## AUTHORS' CONTRIBUTIONS

Yaochen Li: involved in conception and design. Yaochen Li: involved in development of methodology. Yuxian Guo, Junyu Tan, Wei Xiong, Shuzhao Chen and Liping Fan: acquired the data. Yuxian Guo, Junyu Tan, Wei Xiong, Shuzhao Chen, Huada Gene Company, Kangcheng Biology Company and Yaochen Li: analysed and interpreted the data (eg statistical analysis, biostatistics and computational analysis). Yaochen Li: supported the material. Yuxian Guo and Yaochen Li: involved in writing, review and/or revision of the manuscript. Yaochen Li: involved in administrative, study and technical supervision.

## CONSENT FOR PUBLICATION

All authors consent for publication.

## Data Availability

All data and material have been presented within the manuscript and/or additional supporting files.
