# Supplementary figures and images for: Notch3 promotes 3T3‐L1 pre‐adipocytes differentiation by up‐regulating the expression of LARS to activate the mTOR pathway
Source: J Cell Mol Med. 2019 Nov 21;24(1):1116–27. doi: 10.1111/jcmm.14849 (PMC6933334; doi:10.1111/jcmm.14849)

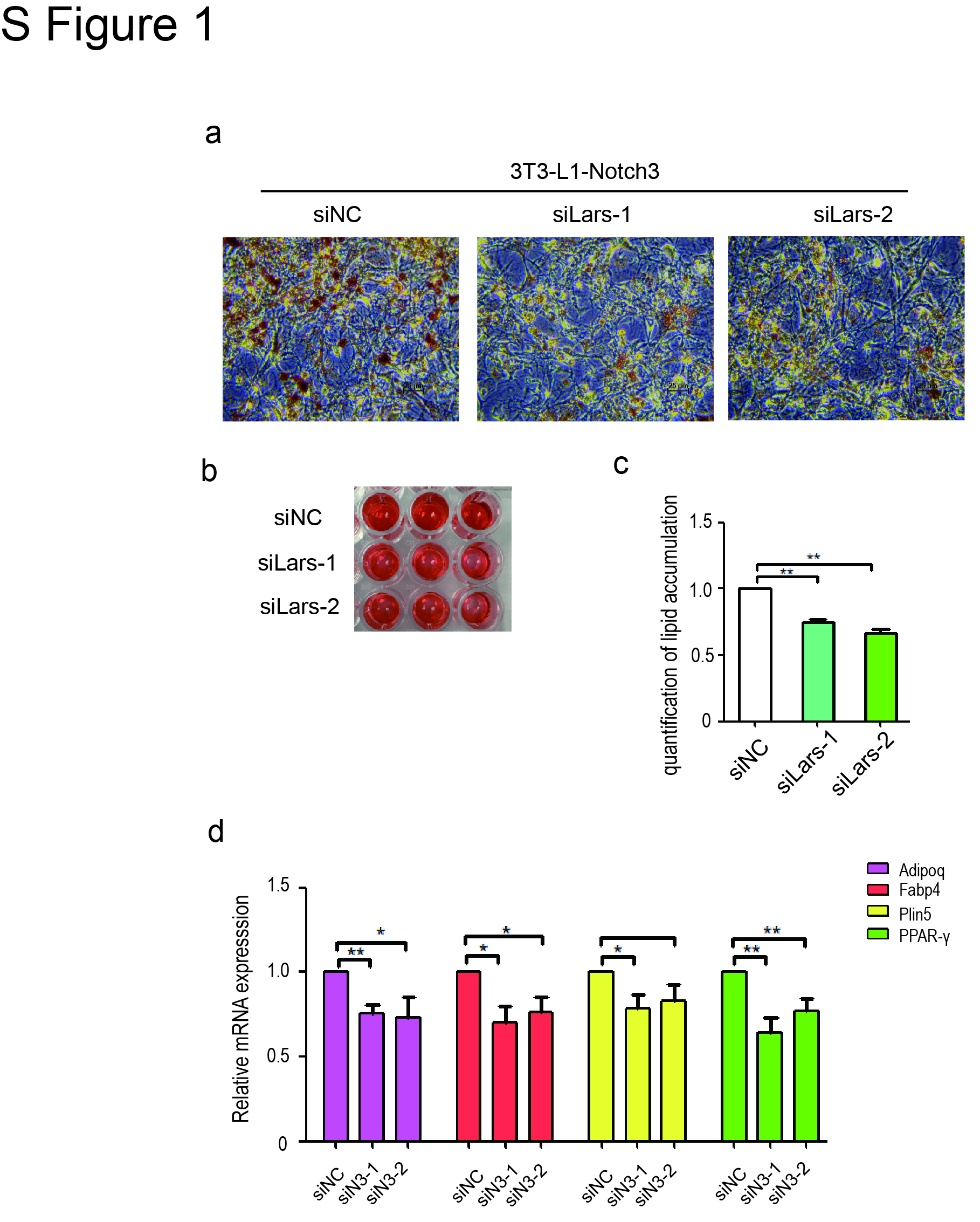

Supplement: Supplementary file 1 [file JCMM-24-1116-s001.tif]

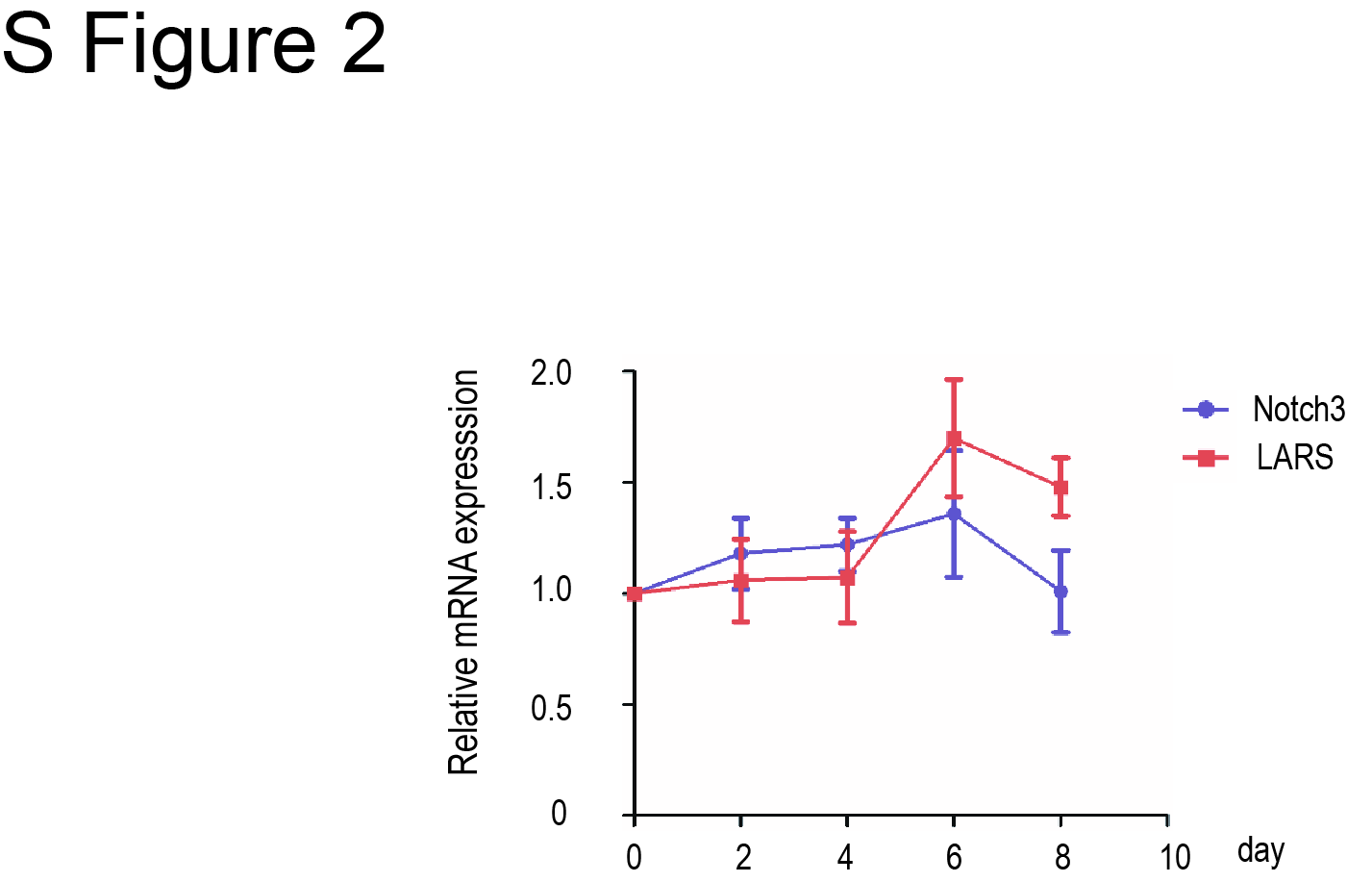

Supplement: Supplementary file 2 [file JCMM-24-1116-s002.tif]
