# Supplementary material for: Notch3 promotes 3T3‐L1 pre‐adipocytes differentiation by up‐regulating the expression of LARS to activate the mTOR pathway
Source: J Cell Mol Med. 2019 Nov 21;24(1):1116–27. doi: 10.1111/jcmm.14849 (PMC6933334; doi:10.1111/jcmm.14849)
Supplement: Supplementary file 3 [file JCMM-24-1116-s003.docx]

**Supplementary Table 1. Primers and interference sequences used in this study**

| Gene | Forward primer | Reverse primer | Purpose |  |  |  |
| --- | --- | --- | --- | --- | --- | --- |
| LARS promoter | 5’- CGAGCTCGAGTTCAACTTAAGGTAA -3’ | 5’- TCCCCCGGGGGACACCAAAGCACAC -3’ | Luciferase Repot assay |  |  |  |
| LARS-ChIP | 5’- AAGCAGTCCATCATGCCGAA -3’ | 5’- CTGAGGCAATCGCAAAGCTC -3’ | ChIP |  |  |  |
| 18S | 5’- AACCCGTTGAACCCCATT-3’ | 5’- CCATCCAATCGGTAGTAGCG-3’ | RT-PCR |  |  |  |
| Notch3 | 5’- CGCTATGCTAGAGCGGATGC -3’ | 5’- AGTGGAGCGGTTCCTGATGA -3’ | RT-PCR |  |  |  |
| LARS | 5’- GAGCAGCAAGGGCAAATACTT -3’ | 5’- ACTGCAAACTCACACTTGGATAA -3’ | RT-PCR |  |  |  |
| MARS | 5’- CTTGAGTCGTCAAAACTGTCCT -3’ | 5’- GTCTGGAACCAACTTTGCAGG -3’ | RT-PCR |  |  |  |
| NARS | 5’- GAGCTGTATGTATCTGACCGAGA -3’ | 5’- AAATGGTGGGAAATGGCTCTTT -3’ | RT-PCR |  |  |  |
| HARS | 5’- GAGGAGCTGGTACGACTCCA -3’ | 5’- GGCGTTTGAAACAGCGGATG -3’ | RT-PCR |  |  |  |
| WARS | 5’- GCTGGAAATGCACCAAAAGATG -3’ | 5’- GCTTGTCCGCACTGTCCAT -3’ | RT-PCR |  |  |  |
| VARS | 5’- GTCCAGCAGTGGGTCAGTTAT -3’ | 5’- GCAGCAGTAAGGCTGTCAC -3’ | RT-PCR |  |  |  |
| Adipoq | 5’- AACATGCCCATTCGCTTTACC -3’ | 5’- TAGGCAAAGTAGTACAGCCCA -3’ | RT-PCR |  |  |  |
| Pck1 | 5’- TTGAGAAAGCGTTCAATGCCA -3’ | 5’- CACGTAGGGTGAATCCGTCAG -3’ | RT-PCR |  |  |  |
| Pparγ | 5’- TACTGTCGGTTTCAGAAATGCC -3’ | 5’- GTCAGCGGACTCTGGATTCAG -3’ | RT-PCR |  |  |  |
| Plin5 | 5’- AAGGCCCTGAAGTGGGTTC -3’ | 5’- GCATGTGGTCTATCAGCTCCA -3’ | RT-PCR |  |  |  |
| Fabp4 | 5’- ACTGGGCCAGGAATTTGACG -3’ | 5’- CTCGTGGAAGTGACGCCTT -3’ | RT-PCR |  |  |  |
| siNC | 5’- UUCUCCGAACGUGUCACGUTT-3’ | 5’- ACGUGACACAGUUCGGAGAATT-3’ | Negtive control |  |  |  |
| siNotch3 -1 | 5’- GCCAGAACUGUGAAGUCAATT -3’ | 5’- UUGACUUCACAGUUCUGGCTT -3’ | interfere Notch3 expression |  |  |  |
| siNotch3 -2 | 5’- CCACGUGUCUUGACCGAAUTT -3’ | 5’- AUUCGGUCAAGACACGUGGTT -3’ | interfere Notch3 expression |  |  |  |
| siNotch3 -3 | 5’- GCACUUUGUGUGAGCGAAATT -3’ | 5’- UUUCGCUCACACAAAGUGCTT -3’ | interfere Notch3 expression |  |  |  |
| siNotch3 -4 | 5’- GCACUUGCCGUGGUUACAUTT -3’ | 5’- AUGUAACCACGGCAAGUGCTT -3’ | interfere Notch3 expression |  |  |  |
| siLars -1 | 5’- GCAAAUACUUUGUCACCUUTT -3’ | 5’- AAGGUGACAAAGUAUUUGCTT -3’ | interfere Lars expression |  |  |  |
| siLars -2 | 5’- GCAGAGCACUGGCUUGAUUTT -3’ | 5’- AAUCAAGCCAGUGCUCUGCTT -3’ | interfere Lars expression |  |  |  |
| siLars -3 | 5’- GGAGUUAAUGGGAGAGGAATT -3’ | 5’- UUCCUCUCCCAUUAACUCCTT -3’ | interfere Lars expression |  |  |  |
