# Supplementary material for: Notch3 promotes 3T3‐L1 pre‐adipocytes differentiation by up‐regulating the expression of LARS to activate the mTOR pathway
Source: J Cell Mol Med. 2019 Nov 21;24(1):1116–27. doi: 10.1111/jcmm.14849 (PMC6933334; doi:10.1111/jcmm.14849)
Supplement: Supplementary file 4 [file JCMM-24-1116-s004.docx]

**Supplementary Table 2. Aminoacyl-tRNA biosynthesis pathway enriched genes**

| PROBE | RANK IN GENE LIST | RANK METRIC SCORE | RUNNING ES | CORE ENRICHMENT |
| --- | --- | --- | --- | --- |
| WARS | 332 | 2.148417234 | 0.053074233 | Yes |
| NARS | 477 | 1.937401533 | 0.108777665 | Yes |
| LARS | 520 | 1.910309553 | 0.16874816 | Yes |
| CARS | 1064 | 1.515148997 | 0.19059101 | Yes |
| VARS | 1203 | 1.442920208 | 0.23052529 | Yes |
| HARS | 1625 | 1.275826812 | 0.2507465 | Yes |
| MARS | 1667 | 1.260649443 | 0.28965178 | Yes |
| IARS | 1726 | 1.241031766 | 0.3270615 | Yes |
| RARS | 2029 | 1.147604942 | 0.3491207 | Yes |
| YARS2 | 2432 | 1.050588131 | 0.36297995 | Yes |
| TARS2 | 2739 | 0.971637905 | 0.37911788 | Yes |
| HARS2 | 2742 | 0.971046984 | 0.41057852 | Yes |
| TARS | 2765 | 0.967490911 | 0.44091427 | Yes |
| GARS | 2776 | 0.964034557 | 0.47174326 | Yes |
| YARS | 3215 | 0.872921109 | 0.47801104 | Yes |
| NARS2 | 3386 | 0.834035933 | 0.49654004 | Yes |
| EPRS | 3675 | 0.782914817 | 0.50745237 | Yes |
| AARS2 | 3783 | 0.765296638 | 0.5269266 | Yes |
| SARS2 | 4147 | 0.703349352 | 0.53146785 | Yes |
| TARSL2 | 4179 | 0.697151423 | 0.55256265 | Yes |
| SARS | 4655 | 0.617352486 | 0.54865646 | Yes |
| MTFMT | 4878 | 0.578861296 | 0.5562674 | Yes |
| SEPSECS | 4916 | 0.572624922 | 0.5730119 | Yes |
| MARS2 | 5019 | 0.557680011 | 0.58599037 | Yes |
| FARSA | 5101 | 0.546349347 | 0.59966034 | Yes |
| FARSB | 5119 | 0.543605745 | 0.616471 | Yes |
| EARS2 | 5133 | 0.541613281 | 0.6334188 | Yes |
| PSTK | 5666 | 0.464301765 | 0.6216615 | No |
| CARS2 | 6259 | 0.383226335 | 0.604241 | No |
| KARS | 6909 | 0.295668632 | 0.581098 | No |
| RARS2 | 6986 | 0.285388172 | 0.58653843 | No |
| AARS | 8306 | 0.12376716 | 0.52399546 | No |
| DARS | 8381 | 0.115378462 | 0.524011 | No |
| PARS2 | 8469 | 0.105766483 | 0.5230581 | No |
| QARS | 9294 | 0.032312416 | 0.48252368 | No |
| VARS2 | 10015 | -0.02928739 | 0.4471395 | No |
| WARS2 | 12656 | -0.137515634 | 0.31837672 | No |
| DARS2 | 12833 | -0.147336766 | 0.3142834 | No |
| IARS2 | 14583 | -0.322463602 | 0.23649785 | No |
| FARS2 | 14950 | -0.367531478 | 0.22997274 | No |
| LARS2 | 16109 | -0.53892827 | 0.18904874 | No |
