# Supplementary material for: Notch3 promotes 3T3‐L1 pre‐adipocytes differentiation by up‐regulating the expression of LARS to activate the mTOR pathway
Source: J Cell Mol Med. 2019 Nov 21;24(1):1116–27. doi: 10.1111/jcmm.14849 (PMC6933334; doi:10.1111/jcmm.14849)
Supplement: Supplementary file 5 [file JCMM-24-1116-s005.docx]

**Supplementary Table 3. Antibodies used in this study**

| Antibody | Cat:# | Company | Con. | Species |  |
| --- | --- | --- | --- | --- | --- |
| Anti-Notch3 | Ab23426 | Abcom | 1:1000 | Rabbit | WB/CHIP |
| Anti-IgG | #3900 | CST | 1:500 | Rabbit | CHIP |
| Anti- LARS | A01109 | BOSTER | 1:1000 | Rabbit | WB |
| Anti- Raptor | 20984-1-AP | Proteintech | 1:1000 | Rabbit | WB |
| Anti- Rictor | NB100-612 | Novus | 1:500 | Rabbit | WB |
| Anti- AKT1/2/3 | BM4400 | BOSTER | 1:1000 | Rabbit | WB |
| Anti- pAKT1(T308) | K006214P | solarbio | 1:2000 | Rabbit | WB |
| Anti-pAkt (Ser473) | #4060 | CST | 1:1000 | Rabbit | WB |
| Anti-GAPDH | TA-08 | ZSGB-BIO | 1:3000 | Mouse | WB |
| Anti- PPAR-gamma | AF6284 | Affinity | 1:1000 | Rabbit | WB |
| Anti- PRDM16 | AF6295 | R＆D | 1:1000 | Sheep | WB |
| Anti- 4EBP1 (Thr37/46) | AF3830 | Affinity | 1:500 | Rabbit | WB |
| Anti- 4EBP1 | AF6432 | Affinity | 1:1000 | Rabbit | WB |
| Anti- pS6K (Thr389/412) | AF3228 | Affinity | 1:500 | Rabbit | WB |
| Anti- S6K | AF6226 | Affinity | 1:1000 | Rabbit | WB |
