# Supplementary material for: Notch3 promotes 3T3‐L1 pre‐adipocytes differentiation by up‐regulating the expression of LARS to activate the mTOR pathway
Source: J Cell Mol Med. 2019 Nov 21;24(1):1116–27. doi: 10.1111/jcmm.14849 (PMC6933334; doi:10.1111/jcmm.14849)
Supplement: Supplementary file 6 [file JCMM-24-1116-s006.docx]

**Supplementary Table 4: Pearson correlation coefficient (R^2^)**

| Sample | siNC-1 | siNC-2 | siNC-3 | siN3-1 | siN3-2 | siN3-3 | siN3-4 |
| --- | --- | --- | --- | --- | --- | --- | --- |
| siNC-1 | 1 | 0.986 | 0.937 | 0.867 | 0.853 | 0.843 | 0.875 |
| siNC-2 | 0.986 | 1 | 0.939 | 0.887 | 0.883 | 0.877 | 0.903 |
| siNC-3 | 0.937 | 0.939 | 1 | 0.812 | 0.819 | 0.811 | 0.846 |
| siNotch3-1 | 0.867 | 0.887 | 0.812 | 1 | 0.983 | 0.986 | 0.989 |
| siNotch3-2 | 0.853 | 0.883 | 0.819 | 0.983 | 1 | 0.985 | 0.984 |
| siNotch3-3 | 0.843 | 0.877 | 0.811 | 0.986 | 0.985 | 1 | 0.989 |
| siNotch3-4 | 0.875 | 0.903 | 0.846 | 0.989 | 0.984 | 0.989 | 1 |
